# An Online Geographic Data Visualization Tool to Relate Preterm Births to Environmental Factors

**DOI:** 10.5888/pcd16.180498

**Published:** 2019-08-08

**Authors:** Marta M. Jankowska, Jiue-An Yang, Jessica Block, Rebecca J. Baer, Laura L. Jelliffe-Pawlowski, Sandra Flores, Tania Pacheco-Warner, Amber Costantino, Jonathan Fuchs, Christina D. Chambers, Gail Newel

**Affiliations:** 1 Calit2/Qualcomm Institute, University of California San Diego, La Jolla, California; 2 California Preterm Birth Initiative, University of California San Francisco, San Francisco, California; 3 Department of Pediatrics, University of California San Diego, La Jolla, California; 4 Department of Epidemiology and Biostatistics, University of California San Francisco, San Francisco, California; 5 Fresno County Preterm Birth Initiative, California State University, Fresno; 6 Central Valley Health Policy Institute, California State University, Fresno; 7 San Francisco Department of Public Health, San Francisco, California; 8 Department of Obstetrics and Gynecology, University of California San Francisco, Fresno Center for Medical Education and Research, Fresno, California

## Abstract

Preterm birth (<37 weeks gestation) continues to be a significant cause of disease and death in the United States. Its complex causes are associated with several genetic, biological, environmental, and sociodemographic factors. Organizing and visualizing various data that may be related to preterm birth is an essential step for pattern exploration and hypothesis generation and presents an opportunity to increase public and stakeholder involvement. In this article, we describe a collaborative effort to create an online geographic data visualization tool using open software to explore preterm birth in Fresno County, where rates are the highest in California. The tool incorporates information on births, environmental exposures, sociodemographic characteristics, the built environment, and access to care. We describe data sets used to build the tool, the data-hosting platform, and the process used to engage stakeholders in its creation. We highlight an important example of how collaboration can increase the utility of geographic data visualization to improve public health and address health equity in birth outcomes.

**Summary**

**What is already known about this topic?**

Preterm birth is a complex health problem with numerous risk factors. Data visualization and mapping of preterm birth and related data are valuable methods of exploring data and engaging the public and stakeholders.

**What is added by this report?**

This project details the process of designing, gathering user feedback, and implementing an online and open source data visualization and exploration tool for preterm birth and related data in Fresno County, California.

**What are the implications for public health practice?**

By giving researchers, stakeholders, and the public free and open source data exploration tools, more informed discussions for reducing preterm birth can occur, and new avenues of research can be explored.

## Preterm Birth and the Need for Data Visualization Tools

Preterm birth (<37 weeks of gestation) contributes significantly to disease and death in the United States, both in the short term and long term. It is associated with higher death rates through infancy and childhood, decreased reproduction, increased risk of having preterm offspring, increased risk of high blood pressure, and symptoms of metabolic syndrome (1–5). The causes of preterm birth are complex and vary for early gestation (20–31 weeks) and late gestation (32–36 weeks) as well as spontaneous (eg, sudden or unplanned preterm birth) and medically indicated (eg, planned and induced preterm birth to minimize other health risks of the baby or mother) subtypes (6). Understanding the causes of preterm birth is vital to informing overarching risk reduction strategies and to developing early detection methods and interventions and can lead to new discoveries in subtype and population-specific risks. However, exploring the myriad of risks for preterm birth — from a woman’s health history, to biomarker data, to behaviors — is challenging for researchers, clinicians, and community health organizations seeking to understand preterm birth and work with women to reduce their risks. These challenges increase as the importance of environment and context become increasingly relevant in preterm birth research and clinical care. Factors such as air pollution, neighborhood environment, and socioeconomic status introduce new data and analytic challenges derived from geographic data formats, which must be integrated with traditional clinical data (7–9). Furthermore, new sources of open-source data are becoming increasingly available, leading to new research and data integration opportunities for better understanding preterm birth (10).

At a population level, health data can be linked geographically through address, census tract, and zip code information. Exploratory spatial data analysis (ESDA) uses geographic linkages to explore patterns, compare nearby geographic regions, and analyze spatial clusters (11), which can indicate underlying place-based variables important to a health outcome, such as crime, pollution, or unexplored environmental factors (12). Visualization of such data is essential for pattern exploration and hypothesis generation. Data visualization offers a field of research and developed tools for exploring patterns, identifying relationships, and synthesizing information in large, multiscale, and multivariate data sets (13). Being able to explore and visualize multilevel and multifactor risks for preterm birth may lead to new mechanistic hypotheses (14), and allow researchers, clinicians, and community health organizations to work with patients in the context of population-level patterns (15). For such a visualization tool to be useful it must be easy to use and immediately accessible, preferably through an online platform; it must leverage ESDA and geographic data science exploratory tools (16) and must have an integrated data structure that includes medical, behavioral, social, and environmental factors.

In this article we discuss the collaborative efforts of several organizations in Fresno County and the state of California to create an online data visualization and exploration tool by using open software (https://delphidata.ucsd.edu/ptbi) to describe preterm birth in Fresno County. We describe data sources and data collection, data features, and mapping functions of the tool. We highlight the utility of online geographic data visualization to explore possible causes of preterm birth and interventions to address it. We also detail how such tools can be built in collaboration with on-the-ground organizations and stakeholders.

## Setting and Partners

Fresno County, California, exemplifies the many challenges presented by the complex and multiple-pathway mechanisms of preterm birth. According to vital statistics for 2007 through 2012 of the California Department of Public Health, Fresno County had the highest overall preterm birth rate in the state, 9.9%, representing 3.7% of California’s preterm births. At a finer geographic scale, 41.4% of all preterm births in that time frame occurred in the south and west-central areas of the city of Fresno. These are the most populated areas of the county, and more than 70% of pregnant women residing there receive Medi-Cal health insurance for prenatal care or delivery on the basis of low-income status. For these reasons, Fresno County is one location of focus for the University of California–San Francisco (UCSF) California Preterm Birth Initiative, a multiyear interdisciplinary research effort with the goal of reducing the prevalence of preterm birth. The Fresno County part of the initiative is a Collective Impact effort that brings together strategic partners from different sectors to focus on the prevention of preterm birth and to address racial and ethnic disparities in its prevalence. Members include local leaders representing public institutions (Fresno County Office of Education, Fresno Housing Authority, Fresno Police Department), public health (Fresno County Department of Public Health; Special Supplemental Nutrition Program for Women, Infants, and Children; First 5 of Fresno), health systems and hospitals (Cal Viva Health, Valley Children’s Healthcare, Community Medical), higher education (Fresno State University, UCSF–Fresno), health clinics (eg, Clinica Sierra Vista), community benefit organizations (eg, AMOR Foundation, Every Neighborhood Partnership), and mothers who experienced preterm birth. Collectively, these groups have committed to address and reduce preterm birth.

A research team from the University of California–San Diego and UCSF composed of geographers, computer scientists, research experts on preterm birth, and a practicing obstetrician was formed in 2016. The team partnered with the Collective Impact effort in Fresno County to begin planning an online geographic data visualization tool that could aid in the assessment, exploration, and discovery of patterns in preterm birth and other social, environmental, and hazard factors. The team met several times with members of the Fresno County Preterm Birth Initiative to obtain feedback about data to include in the tool and the tool’s usability and design.

## Data Inputs and Collection

Finding, formatting, and amalgamating data inputs is one of the largest tasks of any data visualization project. A core goal of this project was to collect data resources that may not be traditionally associated with preterm birth to aid in research discovery and relationship exploration. As a start to the project, various preterm birth researchers were invited to brainstorm variables and data of interest ranging from birth outcomes to environmental factors to supportive resources. From those lists, the core team worked to collect as many data sources as possible (Table). Data were formatted at 2 geographic levels: census tracts, when possible, and Medical Service Study Areas (MSSAs). MSSAs are geographical analysis units defined by the California Office of Statewide Health Planning and Development (OSHPD) and are based on census tracts. Numerous health-related state data sets are provided only at the MSSA unit. Three main types of software were used to amalgamate and format data: ArcMap, version 10.5 (Esri), Microsoft Excel (Microsoft Corp), and SPSS (IBM Corp). All data were manually entered into master CSV (comma-separated values) spreadsheets by geographic unit with data dictionaries. The process included manual data curation decisions, for example, deciding what variables to keep or discard because of redundancy, or methods of aggregation such as counts versus averages.

**Table:** Data Topics, Tool for Visualization of Preterm Birth and Environmental Factors: Variables, Source, and Date Ranges, Fresno County, California^a^

Topic	Indicator Set	Source	Dates
Births	Birth counts, preterm birth by week, spontaneous / indicated	OSHPD, data agreement	2007–2012
Pregnancy indicators	Nulliparity, previous cesarean, previous preterm birth, interpregnancy intervals, prenatal care, using WIC during pregnancy	OSHPD, data agreement	2007–2012
Demographics of women	Race/ethnicity, age, education, place of birth	OSHPD, data agreement	2007–2012
Health of women	BMI, diabetes, hypertension, infection, anemia, mental illness, smoked, drug/alcohol use	OSHPD, data agreement	2007–2012
Preterm birth risk	Risk scores for preterm birth calculated from several variables	OSHPD, data agreement	2007–2012
Care access	Primary care physicians, dental care, psychiatric care	OSHPD, https://oshpd.ca.gov/data-and-reports	2014
	Hospitals, clinics, WIC locations, federally qualified health centers	CHHS, https://data.chhs.ca.gov	2015–2016
Environmental pollution	Ozone, PM2.5, diesel, drinking water contaminations, pesticides, traffic density, clean-up sites, asthma ER visits, overall pollution score	CalEnviroScreen 3.0, https://oehha.ca.gov/calenviroscreen/report/calenviroscreen-30	2010–2014
Demographics of region	Age, race/ethnicity, education, population density, marital status	ACS,^b^ US Census, https://factfinder.census.gov	2007–2012 (ACS), 2010 (Census)
Socioeconomic indicators	Poverty, income, unemployment, housing, crowding, female headed households, car ownership, average house value	ACS,^b^ Census, https://factfinder.census.gov	2007–2012 (ACS), 2010 (Census)
	Crime	ESRI, Business Analyst Data (paid subscription)	2016
	Liquor stores	California Alcohol and Beverage Control, https://www.abc.ca.gov	2012
Cultural indicators	Place of birth, living in same location 1 year ago, diversity index, language spoken	ACS,^b^ https://factfinder.census.gov	2007–2012
Built environment	Bicycle and pedestrian traffic collision	Statewide Integrated Traffic Records System, http://iswitrs.chp.ca.gov	2010–2012
	Walkability	Environmental Protection Agency, https://www.epa.gov/smartgrowth/smart-location-mapping	2010–2012
	Vegetation index, urbanization index, water index	Landsat composite satellite imagery (30m), downloaded and calculated in Google Earth Engine	2010
	Parks	ESRI, http://www.esri.com/data/data-maps	2010–2018
	Transit stops	Fresno County Rural Transit Agency, Fresno Area Express, www.transitwiki.org	2016
	Food access and deserts	United States Department of Agriculture, https://www.ers.usda.gov/data-products/food-access-research-atlas	2015
Health indicators	California Adolescent Sexual Health Needs Index	California Department of Public Health, https://www.cdph.ca.gov	2014
	Child care facilities, elder care facilities, counseling services	CHHS, https://data.chhs.ca.gov	2014–2017
	Immunization rates, newborn deaths, asthma hospitalizations	CHHS, https://data.chhs.ca.gov	2014–2016
	Healthy Priorities Index	Fresno County, http://gis.co.fresno.ca.us/HealthPriorityNDX/	2010–2014

Abbreviations: ACS, American Community Survey; CHHS, California Health and Human Services; ER, emergency room; ESRI, Environmental Systems Research Institute; OSHPD, Office of Statewide Health Planning and Development; PM2.5, particulate matter 2.5 (atmospheric particulate matter with a diameter of less than 2.5 micrometers); WIC, Special Supplemental Nutrition Program for Women, Infants, and Children.

a Topics and indicators from the OSHPD data agreement set include the 81,021 women from the data set. All other indicators are general environmental or population data sets.

b American Community Survey (25).

Data about births were obtained from a birth cohort database maintained by OSHPD. These data are shared through data use agreements and were obtained through collaboration with the California Preterm Birth Initiative under an institutional review board–approved protocol from the California Committee for the Protection of Human Subjects, protocol no. 12–09-0702, ongoing since 2009. The birth cohort file contains detailed information on maternal and infant characteristics derived from linked hospital discharge, birth certificate, and infant death records. Included in the file were all singleton births in Fresno County from 2007 through 2012 with an obstetrician’s best estimate of a gestation at delivery of 20 to 44 weeks and with no known chromosomal abnormalities or major structural birth defects (17). Births were categorized by weeks of gestation based on best obstetric estimates (early preterm birth, gestational age <32 wk; late preterm birth, gestational age 32 to ≤36 wk; term birth, gestational age ≥37 wk) and spontaneous and medically indicated subtypes of birth. Additional data derived from the linked birth certificate and hospital discharge data were race/ethnicity, age, parity, country of birth, pre-pregnancy weight, height, insurance status, smoking, diabetes, hypertension, anemia, maternal education, reported drug use, diagnosed infection, mental illness, and for multiparous women, known number of previous caesarean sections and interpregnancy interval. Data files provided diagnoses and procedure codes based on the International Classification of Diseases, 9th Revision, Clinical Modification (18). The final data set included 81,021 women. Data were aggregated to MSSAs and census tracts with a minimum of 16 women per geographic unit to preserve privacy. In addition to the OHSPD data set of women giving birth, several other indicators were collected from a variety of data sources including the US Census, the Environmental Protection Agency, California Health and Human Services, Fresno County, Esri (Environmental Systems Research Institute), Google, and other California State agencies (Table). Almost all data sets included in the tool are open source and available online, except for data about crime. Not all the indicators align in date with the birth data (2007–2012) because of availability of online data sets. This is a limitation of the tool, which will be improved when newer birth data can be incorporated and as more data are made available online to include past years. Data ingestion is ongoing, and new data sets will be added as they are made available and processed.

## Online Infrastructure and Key Features

The online platform has 2 sides with a user-friendly front end featuring simple click and view options for preterm birth–related topics, and a password protected back end for more advanced users with more data and complex visualization options. Software to develop the tool are all open source framework environments and libraries built with shareability and reproducibility in mind. Using the open source frameworks can allow the tool to be repurposed not only for health but also for other data applications. Code developed for the front-end infrastructures is available online through our GitHub repository (https://github.com/hdscalecollab-ucsd/PTBi-Viz), with back-end code to be added in the near future. We encourage users to use these codes for their own health data visualization projects and to contribute new visualization features back to the repository as they are developed. The front-end development was a direct result of input from stakeholders who, early in the process, voiced a need for a user-friendly and guided data experience for people unfamiliar with data exploration and visualization techniques. Thus, the 2 applications were designed differently to provide their targeted audiences with the data visualization and analytic tools that support knowledge discovery for preterm birth.

The topic-driven front end is intended to create a guided experience to browse data by predefined topics (eg, demographics, environmental pollution) with curated data selected by the team. Each topic has no more than 15 variables per screen, thus limiting the scope of data exploration and making it more manageable for lay users. The data-driven back end application allows users to examine preterm birth–related variables by region and by their relationships with other variables with no limit to the quantity or type of variables included in an analysis. For example, a researcher can explore associations between very preterm birth (between 28 and 32 wk), environmental pollution, socioeconomic disadvantage, and access to care simultaneously.

**Figure 1:**
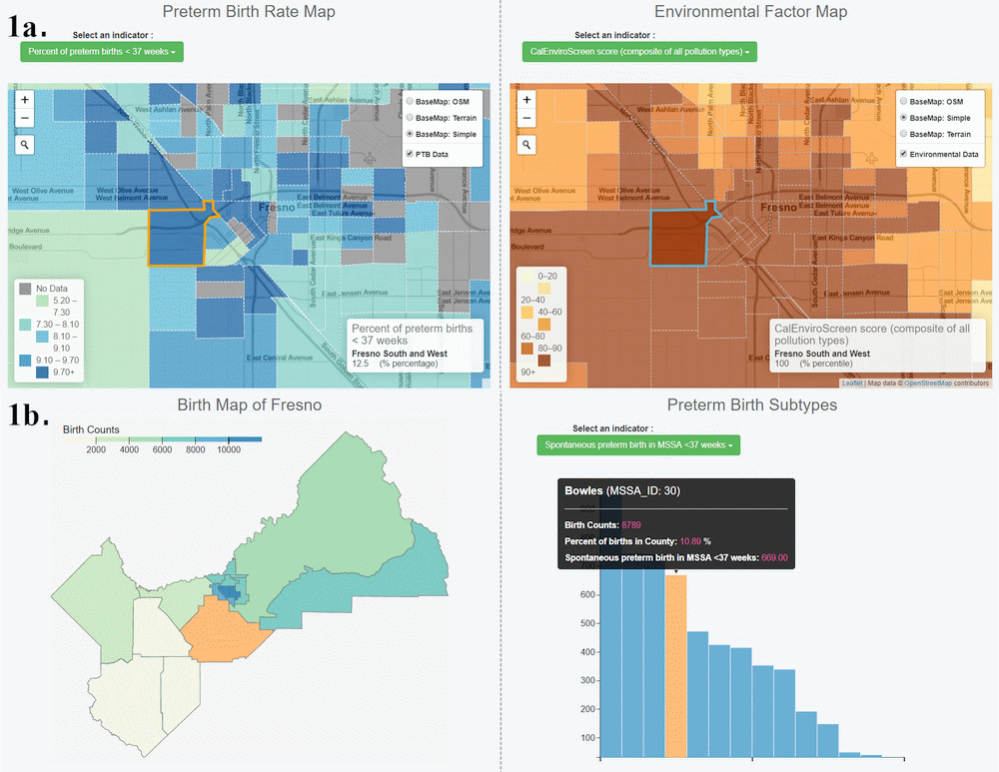
A. Demonstration of the environmental pollution topic of an online geographic data visualization tool for exploring preterm birth in Fresno County, California, using linked maps. Different indicators can be selected for each map. B. The birth topic with overall preterm birth shown on the map and spontaneous preterm birth shown on the indicator histogram. Data elements are linked so that as selection of 1 data value occurs, the location of that data value is displayed on the map.

When logging onto the public site for the first time (https://delphidata.ucsd.edu/ptbi), the user is welcomed with a video briefly explaining the California Preterm Birth Initiative. The 8 topical areas (birth data, health care, demographics, environmental pollution, socioeconomics, pregnancy-related factors, built environment, and health risk) encourage users to begin their exploration of preterm birth and selected geographically organized data. These topics are built with a module tab design pattern where content for each topic is constructed in a separate tab panel and only 1 topic is viewable at a time. When a topic is clicked, the first window to appear in the new page is information about the sources of data being displayed. Within each topic tab, data visualization components are laid out in a side-by-side grid system. These data visualization components are modularized map or graph items that support interactive data visualization by itself or with intercomponent highlighting (across map and graph) and synchronization (between maps). We selected the combination of data visualization components for each topic on the basis of the data type and the spatial resolution of data. For example, pollution data are presented solely with map representations (Figure 1a), whereas birth data include graphical representations of indicators (Figure 1b).

**Figure 2:**
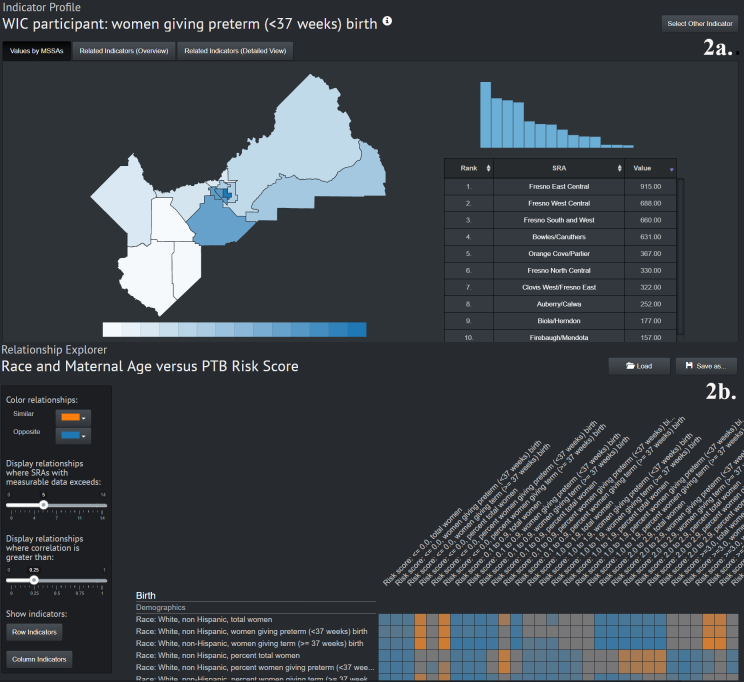
A. Demonstration of the indicator explorer feature of an online geographic data visualization tool to explore preterm birth in Fresno County, California. The indicator explorer feature allows mapping and side-by-side histogram evaluation of any of the hundreds of variables included in the back-end site. As one variable is selected, the relationship between that indicator and others can be viewed in alternate tabs. B. The relationship explorer tab, which builds correlation matrixes for selected indicators. Users can change color, specify the minimum number of geographical units that must be included, and specify the minimum correlation value.

Underlying the back end of the site is the National Science Foundation–supported DELPHI (Data E-Platform Leveraged for Patient Empowerment and Population Health Improvement) developed at UC San Diego, which was implemented to design an asthma management system in San Diego County (19) and can support data-driven public health discoveries from multiple approaches. The site is organized by 4 main visualization functionalities to allow for data exploration by geographic region, by indicator (Figure 2a), by indicator relationships, and by correlation matrixes (Figure 2b). These different functions give the user multiple options to explore data relationships through dynamic pie chart visuals, histograms, ranked associations with other indicators, and heat-map matrix visuals. All functions are linked and highlighted when hovering over them so that as 1 data element or geographical unit is selected, the corresponding units are highlighted. All data are included in the back-end site and are organized by topic area. This organization does require the user to filter through and select variables of interest, but is an important aspect of the data exploration process. In numerous pages the option to save data selections is included so that users can go back and reload previous visualizations. Users can also export various results and outputs into Excel, CSV, and PDF. In future iterations we plan to add ability to download spatial data formats as well.

We used several popular programming framework environments and libraries to develop the applications. Both the front-end and back-end site were developed with the Bootstrap framework (Bootstrap) and the Node.js (Joyent, Inc) environment. The visualization components are built around the D3.js (Mike Bostock) library in the back-end site, and the front-end site mixes up D3.js and its extensions for the chart items and the open-source Leaflet.js (Vladimir Agafonkin) for some mapping features. The data framework transfers processed data between the PostgreSQL (PostgreSQL Global Development Group) database and the visualization platforms through Node.js routes and server-side SQL functions.

## Stakeholder Involvement

The design team followed a user-centered design model for facilitating stakeholder involvement and designing the tool for optimal interface success, which proved successful for designing web-mapping, visualization, and data exploration projects (20–22). We used the user–utility–usability loop developed by Roth, Ross, and MacEachren, in which we collected input and feedback on needs and designs from preterm birth researchers and stakeholders (user), prompting revisions to the conceptualization and functional requirements of the tool (utility), leading to new versions of the data visualization tool (usability) for additional evaluation by our target users, thus restarting the loop (23).

An initial draft of the visualization tool using the DELPHI platform was presented to the Fresno County Preterm Birth Initiative in early 2017. Feedback obtained from stakeholders included comments about variables to be used, geographic resolution, and the need for a more user friendly and simple site that would allow less advanced users to explore and visualize key data sets. The public-facing side of the site was presented again to the Fresno County Preterm Birth Initiative in early 2018 to obtain further input and to narrow down the key topics of interest. The design team then worked with the Fresno County Preterm Birth Initiative Shared Measures Committee, a subgroup of the Fresno initiative that focuses on data and measurement issues, over several meetings to come up with 8 topics to feature on the public-facing side of the tool. The Shared Measures Committee comprises cross-sector leaders and experts in measurement and evaluation and a mother who experienced preterm birth. This committee helps set goals, inform strategies, and establish or develop measures of progress for the Fresno initiative. The iterative feedback between the design team and the Shared Measures Committee was critical for the design of the tool and for determining how the 8 topics should be populated and visualized. Having the design team attend multiple meetings of the Fresno initiative gave additional context to challenges surrounding preterm birth, such as social disadvantage and health care access, and the need to represent such phenomena in maps in a transparent way. Further discussions highlighted how the tool needed to be easy to use for nonprofit organizations to create figures for grant applications and accessible to the public and elected officials. In addition, the tool needed to be bilingual, for both English and Spanish speakers.

The tool was presented to the public and other Fresno initiative stakeholders in July 2018 at the Fresno County Preterm Birth Initiative Forum. The purpose of the event was to convene community members and stakeholders to raise awareness about preterm birth and communicate Preterm Birth Initiative strategies, successes, and challenges. As an example, the team walked through an assessment of environmental pollution as related to preterm birth from the viewpoint of a concerned community member and then from the viewpoint of a public health researcher. Beginning with the front-end, demographics around a neighborhood of interest as related to preterm birth were examined using pie charts and histograms with linked maps (eg, Figure 1b). Moving to the environmental pollution tab, rates of ozone, PM2.5 (atmospheric particulate matter with a diameter of less than 2.5 micrometers), traffic density, and toxic release were examined in relation to 3 neighborhoods in downtown Fresno (eg, Figure 1a). Resulting figures could be used in public presentations, community discussions, or public grant applications. The demonstration then turned to the back-end site where a researcher might want to examine all possible pollution factors in association with not only preterm birth rates, but also mothers’ rates of hypertension, community asthma rates, and poverty indicators by using a correlation matrix (eg, Figure 2b). The tool was presented alongside a poster outlining “Unequal Neighborhoods: Fresno” research on historical zoning and land use policies influencing health disparities. At the event, attendees were encouraged to interact with the tool and with the design team to ask questions and provide feedback. They were given a link to submit further feedback through an online survey and to stay engaged with the project. Feedback is considered and incorporated into the tool design.

## Challenges, Next Steps, and Final Thoughts

The project faced numerous challenges. Data collection, formatting, and updating from various agencies and resources will continue to be a significant task moving forward. The issue of updating data in future years as funding for the project expires is a current topic of discussion for the Fresno County initiative. Our current funding from the California Preterm Birth Initiative supports travel to Fresno and meeting support with stakeholders, a part-time developer, and a part-time–equivalent data-focused researcher. At the very least, a sustainable data updating plan will need to be enacted so that the tool can become a staple feature of the Fresno County Preterm Birth Initiative. As new and updated data are pulled into the tool, the team will also have to face the challenge of demonstrating change from time-based data. The collaborative nature of the project, while an essential feature of making the tool a success, is also not without challenges. When soliciting feedback from stakeholders, the design team had to be cognizant of what was possible in terms of data limitations, the design work involved, and timeline management. Balancing what is possible and what is feasible within a project scope and budget is a challenge for any collaborative project.

The next step in the process will be further dissemination of the tool, because use of the tool by stakeholders thus far has been limited. To this end, the authors are planning a series of workshops focused on local policy makers and staff members, nonprofit organizations, and academic researchers. The tool will also be presented to obstetrics and gynecology residents and students during a UCSF–Fresno grand rounds. The team is also creating a set of how-to videos with examples and trainings to explain how to use tool components and featuring specific use cases such as generating visuals for a grant application or for presentation to a community group, drawing on feedback and previous research showing the need for training across health and geospatial visualization tools (24). We are working to create an Esri story map as an example of how a narrative concerning preterm birth might be created using data on the tool site, and we will also explore open source visualization publishing tools to allow advanced users to create their own story-like narratives from the data visualizations. In the future, a major goal will be to allow users to securely upload and analyze their own collected data sets against the backdrop of public data amalgamated in the tool. We are also in the process of developing similar tools for the San Francisco and San Diego regions.

The Fresno Preterm Birth Initiative’s data visualization tool provides information on births, environmental exposures, sociodemographic characteristics, the built environment, and access to care in a format that makes the information more accessible than it has ever been before. The front-end site design was customized to suit each topic and made as intuitive as possible, allowing community users to engage with preterm birth data, and allowing stakeholders from nonprofit organizations to use figures and data in the tool for grant writing, communication, and policy discussions about preterm birth. The back end offers significantly more data for exploration, along with more powerful tools for data visualization and relationship discovery. The design process, with significant input from the Fresno initiative, preterm birth researchers, and other stakeholders demonstrates the power of working with health and community experts who work daily to improve public health. The original concept of the tool focused solely on using the DELPHI platform and would have been inaccessible to many potential users. Through iterative feedback the design team was able to create a user-friendly front-end site and had significant assistance in identifying and obtaining data sets for inclusion in the tool. Furthermore, because of the collaboration, dissemination of the tool is increased by public events such as the Fresno County Preterm Birth Initiative Forum and publicity of the tool through the initiative’s platforms. Lastly, our feedback to date is unanimously positive. Users of the tool report powerful value for the community at large and express hope to see it grow.
